# Evaluation of the performance of two tuberculosis interferon gamma release assays (IGRA-ELISA and T-SPOT.TB) for diagnosing *Mycobacterium tuberculosis* infection

**DOI:** 10.1016/j.dib.2018.08.112

**Published:** 2018-08-31

**Authors:** Linchuan Wang, Xu-dong Tian, Yan Yu, Wei Chen

**Affiliations:** a The First Affiliated Hospital of Xi’an Jiaotong University, Xi’an, Shaanxi Province, China; b Honghui Hospital, Xi’an Jiaotong University, Xi’an, Shaanxi Province, China

**Keywords:** *Mycobacterium tuberculosis*, IGRA-ELISA, T-SPOT.*TB*

## Abstract

This data contains information from 3727 patients and shows the performance of two IGRAs tests (T-SPOT.*TB* and IGRA-ELISA) *used in China* for screening and diagnostic *Mycobacterium tuberculosis* infection. The positive results were divided into four groups according with the test values, and the proportions of positives in each group were compared. The positive predictive values (PPVs) at different cutoffs for diagnostic active TB and value change trend for the two IGRAs tests were analyzed.

**Specifications Table**

**Table:** 

Subject area	*Biology*
More specific subject area	*Microbiology*
Type of data	*Table and figure*
How data was acquired	LIS and HIS of *the* hospital
Data format	Raw and analyzed data
Experimental factors	Anticoagulant Peripheral blood with heparin Lithium
Experimental features	*IGRA-ELISA* and ELISPOT tests
Data source location	Xi’an, Shaanxi Province, Northwest China.
Data accessibility	Data in the article can be accessible through LIS and HIS of *the* hospital

**Value of the data**

• This work shows the excellent consistency between T-SPOT.*TB* and IGRA-ELISA for diagnosing *Mycobacterium tuberculosis* infection.
• Data in this work demonstrates both T-SPOT.*TB* test and IGRA-ELISA can be used to rule out active TB.
• Data in this work indicates that the value change trend of the IGRA-ELISA was consistent with that of T-SPOT.TB test for the positive samples.

## 1 Data

### 1.1 The performance of the two tests for diagnostic active TB

**Fig. 1:**
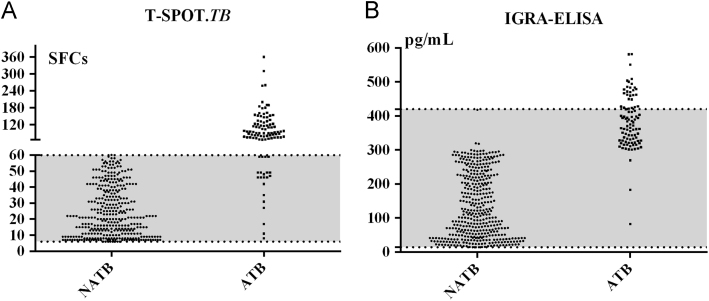
The comparison of values between ATB group and NATB group in positive results for (A) T-SPOT.*TB* test and (B) IGRA-ELISA test. ATB was active tuberculosis; and NATB was nonactive tuberculosis.

The sensitivity and specificity for diagnostic active TB using T-SPOT.*TB* test were 82.9% and 78.6%, and those by IGRA-ELISA were 81.7% and 75.2%, Table 1. The median comparison showed that the *median value in active TB (ATB) group was significantly higher than that in with nonactive TB (NATB) group* for the two IGRAs tests; however, neither of the assays can be applied to diagnose active TB as they cannot differentiate active TB from NATB; see Fig. 1.

**Table 1:** The performance of T-SPOT.TB and IGRA-ELISA for diagnosis ATB.

Performance	Assays		*P* value
	T-SPOT.*TB*	IGRA-ELISA	
Sensitivity:%	82.9	81.7	0.867
Specificity:%	78.6	75.2	0.023*

* Statistically significant association, *P*<0.05.

### 1.2 Analysis of positive test results

**Fig. 2:**
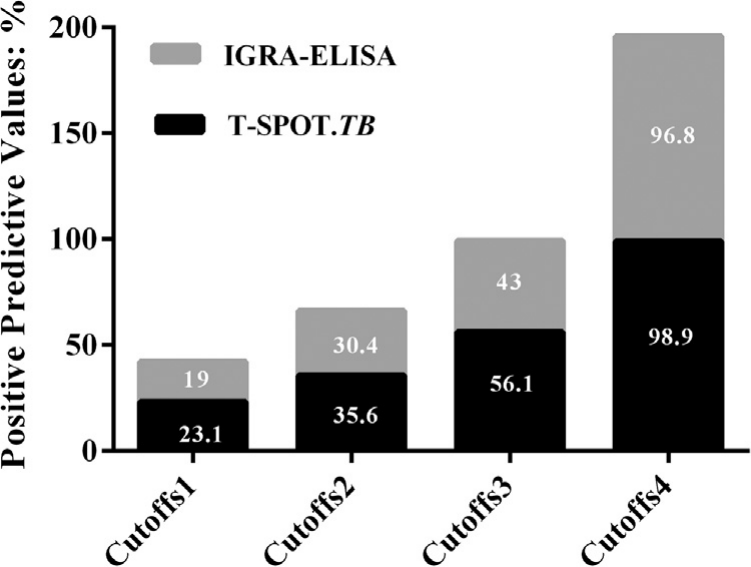
The positive predictive value of T-SPOT.*TB* and IGRA-ELISA test at cutoff 1, cutoff 2, cutoff 3 and cutoff 4. Cutoff 1, 2, 3, 4 for T-SPOT.*TB* were 6, 20, 40 and 60 SFCs; those for IGRA-ELISA were 14, 100, 200 and 300 pg/mL.

The positive results were divided into four groups according with the test values, and except for group 3, the proportion of positives was similarly distributed between the two tests (Table 2). The PPVs of the two tests at different cutoffs for diagnostic ATB were analyzed (Fig. 2).

**Table 2:** The analysis for positive results of T-SPOT.*TB* and IGRA-ELISA tests.

Groups	IGRA-ELISA		T-SPOT.*TB*		*P* value
	Range: pg/mL	Proportions(%)	Range: SFCs	Proportions(%)	
Group 1	14–100	38.06	6–20	39.1	0.232
Group 2	100–200	18.62	20–40	22.46	0.689
Group 3	200–300	24.29	40–60	19.87	0.008*
Group 4	>300	19.03	>60	18.57	0.282

* Statistically significant association, *P*<0.05.

### 1.3 The consistency of the two tests

204 patients were tested using both the *T-SPOT.TB and IGRA-ELISA*. The positive, negative, and total coincidence rates between the two tests were 95.5% (84 of 88), 90.5% (105 of 116), and 92.7% (189 of 204), respectively.There was excellent consistency between the two tests with a kappa value of 0.852 (χ2 = 148.64, *p* < 0.0001). When *84 samples that were positive to both of the two tests* were analyzed, we found that the value change trend of the IGRA-ELISA was consistent with that of T-SPOT.TB test, *and* the two curves intersected at approximately 50 pg/mL (IGRA-ELISA) and 30 SFCs (T-SPOT.TB).

## 2 Experimental design, materials and methods

### 2.1 Enrolled population

A total of 3727 patients with suspected *Mycobacterium tuberculosis* infection at the First Affiliated Hospital of Xi’an Jiaotong University were enrolled. Among them, 204 were tested using both the T-SPOT.TB and IGRA-ELISA, 1794 were tested using the T-SPOT.TB only, and 1729 were tested using the IGRA-ELISA only. The median age was 55 years, the majority of participants (*n*=2100 [56.3%]) were men. 244 patients were diagnosed with active TB if at least one of the following conditions were fulfilled: (i) smear-positive for acid-fast bacilli *or TB PCR-positive*, *n* =19, (ii) chest X-ray findings consistent with a radiological diagnosis of PTB, *n*=53, (iii) granulomata or caseous necrosis observed on histopathological examination of biopsy specimens, *n*=45, or (iv) despite the absence of explicit evidence of active TB on microscopy, *TB PCR*, chest X-ray, or histopathology, the clinical symptoms were consistent with active TB and responded to anti-TB treatment (*n*=127). 129 and 115 of the patients underwent the T-SPOT.*TB* and IGRA-ELISA testing, respectively.

### 2.2 Performing the T-SPOT.TB and IGRA-ELISA test

Peripheral blood samples from the participants were collected in lithium-heparin-coated tubes. The T-SPOT.TB test (Oxford Immunotec Ltd., Abingdon, UK) was performed within 6 h of obtaining the blood sample. The separation and incubation of peripheral blood mononuclear cells were conducted as previously reported [1]. The IGRA-ELISA (WanTai Biological Pharmacy Enterprise Co, Ltd., Beijing, China) was performed within 2 h of obtaining the blood sample. 1 mL of blood was added to each of 3 heparinized tubes (T, N, and P tube). The tubes were incubated at 37 °C for 20–24 h and were then centrifuged at 3000–5000 rpm for 10 min. The supernatant was used for the ELISA assay, performed using an automated instrument.
