# Supplementary material for: Evaluation of the performance of two tuberculosis interferon gamma release assays (IGRA-ELISA and T-SPOT.TB) for diagnosing Mycobacterium tuberculosis infection
Source: Data Brief. 2018 Aug 31;21:2492–5. doi: 10.1016/j.dib.2018.08.112 (PMC6288457; doi:10.1016/j.dib.2018.08.112)
Supplement: Supplementary file 1 — Supplementary material. [file mmc1.doc]

**Conflict of interest**

LW, XDT, YY and WC declare no financial or other conflict of interest.

**Ethics Statement**

The study was deemed exempt from review by the Ethics Committee of the First Affiliated Hospital of Xi'an Jiaotong University because of the retrospective nature of this study.

**Contributors**

LW and YY were major contributors in the writing of the manuscript. LW and WC were responsible for the study design. The statistical analysis and figure of the study were performed by LW, XDT, WC and YY. All authors have read and approved the final manuscript.
